# Profil et antibiosensibilité des bactéries pathogènes associées aux diarrhées chez les patients consultant à l’Hôpital Régional Annexe de Kousseri, Extrême-Nord Cameroun

**DOI:** 10.11604/pamj.2018.29.170.14296

**Published:** 2018-03-23

**Authors:** Jérôme Ateudjieu, Landry Beyala Bita’a, Etienne Guenou, Anthony Njimbia Chebe, Benjamin Azike Chukuwchindun, André Pascal Goura, Anne-Cécile Zoung-Kani Bisseck

**Affiliations:** 1 Département de Santé Publique, Faculté de Médecine et des Sciences Pharmaceutiques (FMSP), Université de Dschang, Ouest Cameroun; 2 Meilleur Accès aux soins de Santé(M.A.SANTE), Yaoundé, Cameroun; 3 Division de la Recherche Opérationnelle en Santé, Ministère de la Santé Publique, Yaoundé, Cameroun; 4 Faculté de Médecine et des Sciences Biomédicales(FMSB), Université de Yaoundé I, Yaoundé, Cameroun

**Keywords:** Profil, antibiosensibilité, bactérie, diarrhée, Profile, antibiotic susceptibility, bacteria, diarrhea

## Abstract

**Introduction:**

Du fait de l'accès limité au laboratoire au Cameroun, la prise en charge des cas de diarrhée dans la plus part des formations sanitaires est basée sur le diagnostic de présomption. L'objectif de notre étude était de déterminer la distribution et la sensibilité aux antibiotiques habituellement prescrits contre les bactéries pathogènes associés aux diarrhées à l'Hôpital Régional Annexe de Kousseri(HRAK) de Juillet à Octobre 2015.

**Méthodes:**

Il s'agissait d'une étude descriptive et transversale ciblant toute personne consentante consultant pour diarrhée à l'HRAK pendant la période d'étude. De chaque patient était collectés un échantillon de selles et les données par questionnaire anonyme administré en face à face. Chaque échantillon de selles était cultivé sur milieu spécifique aux enterobactéries et analysé suivant la méthode standard de coproculture. La sensibilité des souches isolées aux antibiotiques fréquemment prescrits, a été évaluée et les proportions des patients présentant chaque germe pathogène et de germe sensible à chaque antibiotique étaient estimées.

**Résultats:**

Au total 45(30,0%) des 150 cas de diarrhée inclus étaient associés à une bactérie enteropathogéne dont 37(82,2%) chez les enfants de 0 à 5 ans. *Escherichia coli* était la bactérie la plus représentée avec 30 cas(66%) suivis des cas de *Salmonella spp*, 7(16%); Vibrio spp, 5(11%); *Aeromonas spp*, 2(4%) et *Shigella spp*, 1(2%). Les antibiogrammes réalisés ont montré que 17(56,7%), 14(46,7%) et 5(16,7%) *E. coli* étaient sensibles à la Ciprofloxacine, Ceftriaxone, au Cotrimoxazole respectivement. 4(57,14%), 2(28,57%) *Salmonella spp.* était sensible au Ceftriaxone et au cotrimoxazole respectivement.

**Conclusion:**

Près du tiers des cas de diarrhée consultant à l'HRAK en saison de pluie sont associés à au moins une bactérie pathogène. La sensibilité des germes isolés aux antibiotiques couramment prescrits reste très limitée. Dans les formations sanitaires de l'Extrême Nord Cameroun ou le personnel de santé est obligé de faire des prescriptions d'antibiotiques contre les diarrhées sur la base des diagnostics de présomption, un système de surveillance des germes associés et de la sensibilité de ceux-ci aux antibiotiques prescrits en routine devra être mis en place.

## Abstract (en)

**Introduction:**

In most of the health facilities in Cameroon, the management of patients with diarrhea is based on presumptive diagnosis due to limited laboratory resources. This study aimed to determine germs profile and their susceptibility to antibiotics usually prescribed against pathogenic bacteria associated with diarrheas at the Kousseri Regional Hospital Annex from July to October 2015.

**Methods:**

We conducted a descriptive and cross-sectional study of all consenting individual presenting with diarrhea to the Kousseri Regional Hospital Annex during the study period. Stool samples were collected from each patient. Patient data were collected via anonymous questionnaire with face to face interview. Each stool sample was grown on media for enterobacteria and analyzed following the standard stool culture method. The sensitivity of the isolated strains to the most frequently prescribed antibiotics was assessed and the proportions of patients with each pathogen germ and of germ sensitive to each antibiotic were estimated.

**Results:**

Out of 150 patients with diarrhea included in the study 45(30.0%) had enteropathogenic bacteria, of which 37(82.2%) in children aged 0-5 years. Escherichia coli was the the most common bacterium(30 cases, 66%) followed by Salmonella spp(7 cases, 16%), Vibrio spp,(5 cases, 11%), Aeromonas spp(2 cases, 4%), and Shigella spp(1 cases, 2%). Susceptibility tests were performed which showed that 17(56.7%), 14(46.7%) and 5(16.7%) E. coli were susceptible to ciprofloxacin, ceftriaxone, cotrimoxazole respectively; 4(57.14%), 2(28.57%) Salmonella spp. were susceptible to ceftriaxone and cotrimoxazole respectively.

**Conclusion:**

Nearly a third of the cases of diarrheas at the Kousseri Regional Hospital Annex in the season of rain were associated with at least a pathogenic bacterium. The susceptibility of isolated germs to commonly prescribed antibiotics was very limited. Health staff in the facilities in the extreme north of Cameroon prescribe antibiotic against diarrheas on the basis of presumptive diagnosis. Hence, the necessity to implement a system for the monitoring of the associated germ profile and of their susceptibility to commonly prescribed antibiotics.

## Introduction

Les maladies diarrhéiques représentent un problème de santé majeur dans le monde, surtout dans les pays en développement où elles sévissent à l'état endémique [1]. Deuxième cause de mortalité chez l'enfant de moins de cinq ans, la diarrhée est à l'origine de 760.000 décès d'enfants par an et l'on recense environ 1,7 milliard de cas de diarrhées chaque année dans le monde [2]. Au Cameroun, la diarrhée infantile constitue l'une des principales causes de décès chez les enfants de moins de 5 ans, après le paludisme, la rougeole et les maladies des voies respiratoires [3,4]. Le système de santé du Cameroun tout comme l'Hôpital Régional Annexe de Kousseri (HRAK) n'ont jusqu'à présent pas encore mis à la disposition du personnel de soins un guide de prise en charge des diarrhées [4, 5]. De même, dans plus des quatre cinquième des formations sanitaires fonctionnelles de la région de l'extrême Nord Cameroun, il manque soit le microscope, soit le personnel ou une source d'énergie pour offrir l'examen des selles à la demande. Il en résulte que les prescriptions de médicaments répondant aux cas de diarrhées ou le suivi des patients sous traitement sont basées uniquement sur les arguments cliniques. Cette approche peut apporter des soins adéquats à un certain nombre de malades mais reste limitée car elle peut soit entrainer des résistances aux médicaments prescrits; soit exposer les patients à des traitements non justifiés; soit retarder ou rater la prise en charge adéquate des cas de diarrhées. L'une des réponses efficientes à cette limite est la surveillance du profil de germe et de la sensibilité aux antibiotiques et l'utilisation des résultats de cette surveillance pour orienter la prise en charge des cas de diarrhées dans chacune des régions sanitaires du Cameroun. La présente étude a été conduite d'une part pour évaluer la faisabilité de la mise en place d'un tel système de surveillance dans un hôpital qui a le minimum de plateau technique pour conduire une telle activité et d'autre part déterminer le profil des germes associés aux diarrhées et leur sensibilité aux antibiotiques à l'HRAK. Cette formation sanitaire a été choisie puisqu'elle reçoit en même temps les patients venant de la ville de Kousseri, de N'Djaména la capitale du Tchad qui est un pays voisin du Cameroun et les cas référés des formations sanitaires de quatre districts de santé totalisant en 2015, une population générale estimée à sept cent mille âmes.

## Méthodes

Avant sa mise en œuvre, ce protocole a été approuvé par le comité national d'éthique de la recherche pour la santé humaine (CNERSH) du Cameroun. La référence de la clairance éthique délivrée à cet effet est la suivante: N°2015/08/636/CE/CNERSH/SP du 13 Août 2015.

**Schéma d'étude:** il s'agissait d'une étude descriptive de type transversal qui a impliqué: la détection hospitalière des cas de diarrhée, l'identification et le test de sensibilité aux antibiotiques couramment prescrits par le personnel de santé des bactéries associées.

**Figure 1:**
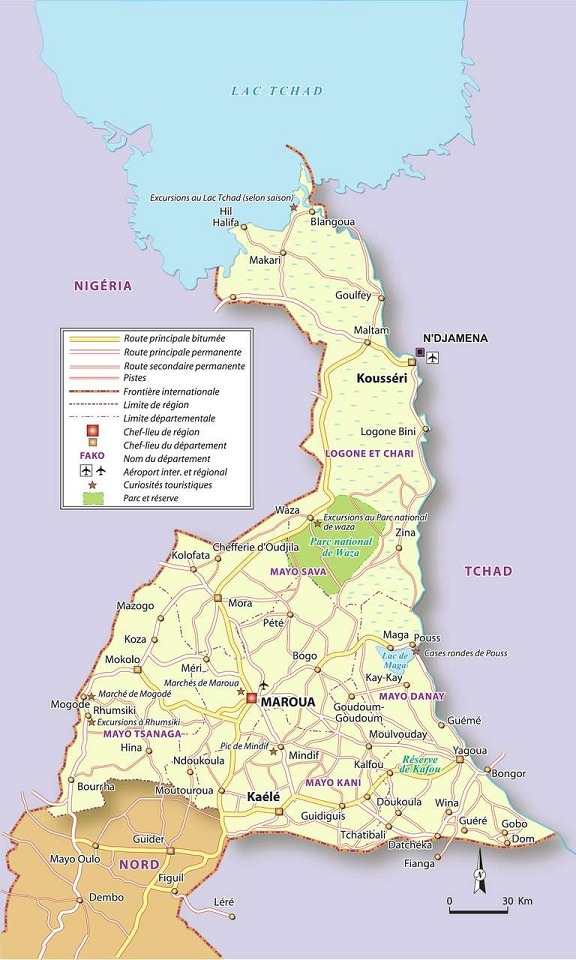
Ville de Kousséri dans la région de l'Extrême Nord Cameroun

**Site et période de l'étude:** Cette enquête était menée dans la région de l'extrême Nord du Cameroun précisément dans la ville de Kousseri; où les épidémies de choléra sont récurrentes et où l'incidence des maladies diarrhéiques est de 31,2% selon l'EDS-MICS, 2011[3]. L'étude s'est déroulée du 24 Juillet au 23 Octobre 2015. Les patients inclus provenaient des services de consultation et d'hospitalisation de l'Hôpital Régional Annexe de Kousseri (HRAK) (Figure 1). Les échantillons de selles prélevés de ces patients ont été préparés et testés au Laboratoire de Surveillance du Choléra et des Maladies Diarrhéiques (LSCMD) basé à l'HRAK, mis en place par l'ONG MA.SANTE (Meilleur Accès aux soins de santé) avec l'appui du projet DOVE (Delivering Oral Cholera Vaccine Effectively) basé à l'Université Johns Hopkins aux USA.

**Sélection des participants:** Les patients de tout âge sans distinction de sexe et de provenance ayant fait au moins trois selles au cours des 24 heures précédant leur consultation à l'HRAK étaient éligibles pour participer à l'étude. Tous les patients éligibles étaient consécutivement sollicités pendant la période de l'étude. Chacun d'eux ou son parent légal était abordé par l'enquêteur en charge, informé de l'étude et invité à participer. Ceux qui consentaient signaient la fiche de consentement, recevaient un questionnaire et un échantillon de selle était collecté de chacun d'eux.

**Procédure de collecte des données et des échantillons:** Les données étaient collectées à l'aide d'un questionnaire prétesté dans une formation sanitaire voisine administré en face à face par l'enquêteur. Les principales variables collectées étaient les caractéristiques sociodémographiques des participants, les antécédents médicaux, les caractéristiques cliniques des patients, macroscopie des selles, microscopie à l'état frais et après coloration au Gram, les caractéristiques de la culture et les résultats de l'antibiogramme. Chaque patient inclus, était prélevé un échantillon de selle. Ce prélèvement était effectué soit sur la selle remise par le patient par une spatule stérile et déposé dans un pot stérile et quand le patient ne pouvait pas faire des selles par écouvillonnage rectal et gardé stérile dans le capuchon de l'écouvillon.

**Transport, traitement et analyse des échantillons prélevés:** Le pot ou l'écouvillon était acheminé immédiatement au LSCMD et immédiatement traité en trois temps: examen macroscopique, examen microscopique direct et après coloration au Gram enfin réalisation de l'antibiogramme. Au laboratoire, pendant l'examen macroscopiquement, la consistance, la couleur, la présence ou non de glaire, du sang ou de mousse étaient notées. Examen microscopique direct de la selle était fait à l'eau physiologique et au lugol au grossissement 10 puis 40X à la recherche de parasites et des bactéries mobiles. Après l'échantillon était coloré au Gram pour apprécier la cytologie, la coproflore: « selles normales: 30% de Gram +, 70% de Gram -»; rechercher un dysmicrobisme (flore monomorphe à Gram + ou -); Culture et isolement: nous réalisions une culture directe et une culture indirecte sur chaque échantillon de selles. Chaque échantillon de selles était directement ensemencé sur Hektoen pour les patients dont l'âge était supérieur a deux ans et sur EMB (Eosine Métylen Blue) pour les patients dont l'âge était inférieur à deux ans. La culture indirecte consistait à inoculer tous les échantillons dans le milieu d'enrichissement Muller Kauffman puis incuber à 37°C pendant 6 heures et après cette incubation, nous ensemencions un prélèvement issu du Muller Kauffmann dans Hektoen en utilisant la technique de quadrants [6]. L'identification des souches a été faite en utilisant la galerie API 20E (BioMéreux) après un test biochimique d'orientation à la recherche des cytochromes oxydases grâces aux disques Oxydase (HIMEDIA^®^); Réalisation de l'antibiogramme: La sensibilité des bactéries isolées était déterminée en utilisant la méthode de diffusion des disques d'antibiotiques sur gélose. Le choix des antibiotiques à tester était fait sur la liste publiée par le Comité de l'Antibiogramme de la Société Française de Microbiologie(CASFM) [7]. Dans cette liste, nous avons choisi les antibiotiques en fonction des habitudes de prescription des ATB par le personnel de santé de la zone d'étude [8]. Ainsi les antibiotiques testés lors de la réalisation de l'antibiogramme était constitué de: - β-lactamines: Pénicillines(Amoxycilline, Amoxycilline+ac.clavulanique); Céphalosporines: Ceftriaxone; Carbapénèmes: Imipènème; - Les quinolones et fluoroquinolones (acide nalidicique, ciprofloxacine); - Les cyclines: tetracycline, - Les sulfamides et associations: trimethroprime-sulfamethonazole - Les phénicolés: chloramphenicol; - Les Aminosides: Gentamicine, Amikacine; - Les macrolides: Erythromycine; - Polypeptides: colistine. Ces antibiotiques subissaient un contrôle de qualité avant leur utilisation. En effet, pour évaluer la validité des disques et la conformité du milieu Muller Hinton (MH), des souches de références ont été utilisées notamment Escherichia coliATCC 25922. Nous avons testé la sensibilité de cette souche de référence vis-à-vis des différents disques d'antibiotiques sélectionnés pour notre étude. Les diamètres obtenus étaient comparés aux diamètres standards recommandés par le CASFM. La technique d'ensemencement de l'inoculum était celle d'écouvillonnage sur gélose telle que décrite par Kirby et Bauer.

**Traitement et analyse des données:** Les données recueillies ont été saisies et analysées sur micro-ordinateur avec le logiciel Epi Info version 3.5.3 et Microsoft Excel 2007. Les principaux indicateurs estimés étaient les proportions des caractéristiques sociodémographiques des participants, des antécédents médicaux, des caractéristiques cliniques des patients, les caractéristiques macroscopiques des selles, les proportions des germes parasitaires et bactériens et les résultats de l'antibiogramme.

## Résultats

**Caractéristiques sociodémographiques des patients:** Des 160 patients diarrhéiques sollicités, 150(93,2%) ont acceptés de participer. La tranche d'âge la plus représentée était celle de 0 à 5 ans avec 120/150(80,0%) de cas. Les personnes interviewées étaient majoritairement non scolarisées 94(62,7%) et au niveau scolaire primaire 18(12,0)%. La majorité de personnes incluses étaient sans emploi dont 119(79,3%). Le district de santé de Kousseri était le plus représenté avec 132(88,0%) cas (Tableau 1).

**Tableau 1:** Distribution des caractéristiques sociodémographiques des patients

Variables	Effectifs (n)	Pourcentage (%)
**Tranche d’âge (ans)**		
**0-5**	**120**	**80**
5-15	12	8
15 et plus	18	12
**Total**	**150**	**100**
**Genre**		
**Homme**	**94**	**62,7**
Femme	56	37,3
**Total**	**150**	**100**
**Niveau d’instruction**		
**Non scolarisé**	**94**	**62,7**
École islamique	16	10,7
Primaire	18	12,0
Secondaire	18	12,0
Supérieur	4	4,7
**Total**	**150**	**100**
**Profession**		
**Sans emploi**	**119**	**79,3**
Secteur informel	27	18,0
Secteur formel	4	2,7
**Total**	**150**	**100**
**District de provenance**		
**Kousseri**	**132**	**88**
Mada	5	3,3
Makary	4	2,7
Goulfey	3	2,0
Autres (Pays voisins)	6	4,0
**Total**	**150**	**100**

**Caractéristique de la diarrhée:** La date de début de la diarrhée était en moyenne de 7 jours avec des extrêmes de 1 à 40 jours. Les selles collectées étaient aqueuse chez 110/150(73,3%) participants, glaireuses chez 21/150(14,0%) participants et sanguinolente chez 6/150(4,0%) participants.

**Signes et affections associés:** La fièvre était le symptôme le plus souvent associé avec 93/150(62,0%) des cas suivis des vomissements 75/150(50,0%). La malnutrition était l'affection la plus fréquemment associée à la diarrhée avec 54/150(36,0%) surtout chez les enfants de 0 à 5 ans (Tableau 2).

**Tableau 2:** Signes et affections associés à la diarrhée

Signes et symptômes	Nombre de cas	Pourcentages (%)
**Fièvres**	**93**	**62,0**
Vomissements	75	50,0
Douleur abdominale/Ballonnement abdominal	41	27,4
Déshydratation	30	20,0
Asthénie	10	6,7
Nausée	5	3,4
Ténesme	1	0,7
Toux	4	2,7
**Affections**		
**Malnutrition**	**54**	**36,0**
VIH/SIDA	4	2,7

### Résultats étiologique

**Agents isolés:** Des cent cinquante(150) patients qui ont bénéficiés d'une coproculture, 100(66,6%) ont présentés une culture positive avec 45/150(30,0%) bactéries pathogènes dont 37/120(30,8%) chez les enfants de 0 à 5 ans. Les bactéries pathogènes identifiés étaient, *Eschérichia coli* chez 30/45(66,6%) patients, *Salmonelle Spp.* chez 7/45(15,5%) patients, des Vibrio Spp. chez 5/45(11,1%) patients et des *Aeromonas sobria*chez 2/45(4.4%) patients (Tableau 3). Le germe le plus fréquement isolé chez les enfants de moins de 5 ans était E. coli chez 27/37(73,0%). Les 55/100(55,0%) non pathogènes isolés incluaient: le groupe K.E.S (Klebsiella Spp., Enterobacter Spp. et Serratia Spp.) isolés chez 15/55(27,3%), 10/55(18,18%), et 17/55(30,9%) patients respectivement, *Proteus mirabilis* chez 7/55(12,7%) patients, Citrobacter Spp. chez 4/55(7,3%) patients, *Kluyvera Spp. et Pseudomonas aeruginosa* chez 1/55(1,8%) patient respectivement (Tableau 4).

**Tableau 3:** Répartitions de bactéries pathogènes isolées

Bactéries isolés	Fréquences	Pourcentage (%)
*Aeromonas sobria*	2	4,44
***Escherichia coli***	**30**	**66,7**
*Salmonella spp*	7	15,55
*Shigella spp*	1	2,22
*Vibrio spp*	5	11,11
Total	45	100

**Tableau 4:** Répartition des bactéries non entéropathogènes isolés

Bactéries isolés	Fréquences	Pourcentage (%)
*Citrobacter spp*	4	7,27
***Enterobacter spp***	**10**	**18,18**
***Klebsiella spp***	**15**	**27,27**
*Kluyvera spp*	1	1,81
*Proteus mirablis*	7	12,72
*Pseudomonas aeruginosa*	1	1,81
***Serratia spp***	**17**	**30,90**
Total	**55**	**100**

**Sensibilité des bactéries pathogènes aux antibiotiques:** Huit 8 grandes familles d'antibiotiques ont été testées. Les résultats sont présentés dans le tableau 5. Il ressort que seul 16,7% et 56,7% d'*E. coli* qui est le germe pathogène le plus fréquemment associé aux diarrhées sont respectivement sensibles au cotrimoxazole et ciprofloxacine qui sont les antibiotiques les plus prescrits en cas de suspicion de diarrhée bactérienne. Les bactéries *Aeromonas, E. coli, Salmonella spp, Shigella et Vibrio spp.* étaient à 100% résistant à l'amoxiciciline et amoxiciline + acide clavulanique. De même, E. coli était résistant à la ceftriaxone (53.3%), la chloramphénicole (50%), la tétracyclline (83,3%), l'erythromicine (100%), l'acide nalidixique (69.5%), la ciprofloxacine (43,3%), la cotrimoxazole (83.33%) et à la colistine(100%) (Tableau 5).

**Tableau 5:** Répartition générale de sensibilité des agents bactériens isolés

Entéropathogènes DCI des Antibiotiques testés	Aeromonas sobria (2)			Escherichia coli (30)			Salmonella spp (7)			Shigella spp (1)			Vibrio spp. (5)		
	S(%)	I(%)	R(%)	S(%)	I(%)	R(%)	S(%)	I(%)	R(%)	S(%)	I(%)	R(%)	S(%)	I(%)	R(%)
Amoxycilline 30 µg (AX)	0	0	100	0	0	100	0	28,57	71,43	0	0	100	0	0	100
Amoxycilline+ac.clav ( 20/10) µg (AMC)	0	0	100	0	0	100	0	28,57	71,43	0	0	100	0	0	100
**Ceftriaxone 30µg (CTX)**	**100**	**0**	**0**	**46,7**	**10**	**43,33**	**57,14**	**0**	**42,86**	**100**	**0**	**0**	**40**	**20**	**40**
Imipénème 10 µg (IMI)	100	0	0	85	5	10	75	0	25	100	0	0	33,33	0	66,7
Gentamicine 15µg (10UI) (GM)	50	50	0	65	5	30	50	0	50	0	0	100	75	0	25
Amikacine 30µg (AN)	100	0	0	90	5	5	100	0	0	100	0	0	66,7	0	33,33
Chloramphénicol 30µg (C)	100	0	0	50	26,7	23,33	57,14	14,28	28,57	100	0	0	80	20	0
Tétracycline 30 µg (TE)	0	0	100	16,7	0	83,33	0	14,28	85,71	0	0	100	0	0	100
Erythromycine 15 µg (E)	0	0	100	0	0	100	0	14,28	85,71	0	0	100	0	0	100
Acide nalidixique 30 µg (NA)	100	0	0	30,43	21,7	47,82	75	25	0	0	0	100	0	0	100
**Ciprofloxacine 30 µg (CIP)**	**100**	**0**	**0**	**56,7**	**0**	**43,33**	**71,42**	**0**	**28,57**	**0**	**0**	**100**	**40**	**0**	**60**
**Sulfaméthoxazole (Cotrimoxazole) 25µg (SXT)**	**100**	**0**	**0**	**16,7**	**0**	**83,33**	**28,57**	**0**	**71,42**	**0**	**0**	**100**	**20**	**20**	**60**
Colistine 50 µg (CL)	0	0	100	0	5	95	0	25	75	0	0	100	0	0	100

S: sensible; I: intermédiaire; R: résistant

**Profil des parasites et mycoses isolés:** Chez quatre vingt-huit 88/150(58,6%) patients consultant pour diarrhée dont 66/120(55,0%) enfant de moins de 5 ans, les parasites ont été isolés. Il s'agissait essentiellement d'Entamoeba histolyticae dans 66/88(75,0%). Les éléments en forme de levure ont été isolés chez 77/150(51,3%) dont 70/120(58,3%) chez les enfants de moins de 5 ans. Des associations Entamoeba histolyticae-éléments lévuriformes ont été observé chez 34/150(22,6%) patients.

**Évolution des patients hospitalisés:** Deux cas de décès ont été enregistrés dans le service de pédiatrie chez les 100 patients hospitalisés. Ils étaient âgés respectivement de 11 et 18 mois de sexe masculin et présentaient un syndrome cholériforme(Plus de 10 selles en 24 hrs, déshydratation plan “C”) et ayant été diagnostiqué « une malnutrition aigüe sévère »; *E. coli* a été le germe isolé chez ses patients. L'évolution a été favorable chez 88 patients et n'était pas connue chez 10 patients « évadés ».

## Discussion

Cette étude nous a permis de déterminer le profil des bactéries le plus souvent associé aux cas de diarrhées chez les patients consultants dans un le seul hôpital de référence de la région du Lac Tchad Cameroun et d'évaluer la sensibilité de bactéries isolées aux antibiotiques habituellement prescrits par le personnel de santé de cette zone. La connaissance de la distribution par tranche d'âge des étiologies infectieuses des diarrhées est d'une importance capitale pour guidés le choix des types d'anti-infectieux à proposer chez un patient présentant ce symptôme. Les résultats de la présente étude indiquent qu'au moins une bactérie pathogène a été isolée chez un patient sur trois présentant une diarrhée et qu'un parasite et une mycose ont été identifiés chez plus de la moitié des cas de diarrhée. Ces proportions n'ont pas significativement varié de l'adulte à l'enfant. Cette distribution est différente de celle observés dans d'autres études faites dans d'autres pays africain et de la distribution générales connues en Afrique [8-12]. Cette différence ne devrait pas être une curiosité puisque plusieurs études ont démontré que la distribution par groupe d'âge et des germes associés est fonction de l'environnement, des habitudes en termes d'hygiène, de l'accès de l'enfant à l'allaitement maternelle et à la vaccination [4, 13-15]. Ceci est contraire à la distribution livresque et standardisées des étiologies de diarrhée par tranche d'âge et souligne l'obligation de renseigner cette distribution sur la base des études périodiques et ou sur une surveillance épidémiologiques.

La disponibilité de l'information sur les germes isolés chez les patients diarrhéiques permet d'orienter les prescriptions. Dans la présente étude, les bactéries pathogènes, les parasites et les mycoses ont été retrouvés chez 30,0%, 58,7% et 51,3% respectivement. Étant donné que la présence de certaines formes de parasites comme les Kystes d'*Entamoeba* et de certaines formes de levures déterminées n'est pas considéré comme une cause irréfutable de la diarrhée, nous ne pouvons affirmer que la présence de ses germes avait une relation de causalité avec les diarrhées investiguées. Cette situation se conforte par le fait que les causes virales qui sont connues dans la littérature comme étant les plus fréquentes n'ont pas été investiguées. Dans certaines études, cette situation a été clarifiée en conduisant une étude cas témoins dans laquelle les mêmes germes étaient recherchés chez les cas constitués de personnes présentant la diarrhée et les témoins constitués des personnes appariés sans diarrhées [9]. Ce schéma a permis d'estimer et comparer la probabilité de survenues d'un germe suspect pathogène entre les groupes des cas de diarrhée et ceux des témoins. Nous suggérons et allons adopter ce schéma pour investiguer les étiologies de diarrhée dans notre contexte ou la relation de causalité entre un germe et le symptôme diarrhée est difficile à établir.

Le profil des bactéries associées aux diarrhées est une information indispensable pour orienter les prescriptions d'antibiotique quand une bactérie pathogène est suspectée comme cause de la diarrhée. Dans la présente étude, *Escherichia coli, Salmonelle Spp., Vibrio Spp. et Aeromonas sobria* sont des bactéries suspectes pathogènes qui ont été identifiés chez les participants de la présente étude. Comme dit plus haut, le contexte de la présente étude ne permettait pas qu'il soit établi la relation de causalité entre ces cas de diarrhées et les bactéries isolées. Des études conduites dans d'autres contextes ont décrit un profil de bactéries différent de celui de la présente étude [11, 16-21]. Cette différence pouvant être expliquée par les différences d'environnements, de comportement et de biologie des patients. La sensibilité aux antibiotiques a absolument besoin d'être surveillée dans un environnement où la prescription des antibiotiques est habituellement faite sur des bases présomptives. La présente étude a testé l'amoxicilline, le cotrimoxazole, la ciprofloxacine et le ceftriaxone qui affirmés prescrits par le personnel de santé en cas de suspicion de diarrhée infectieuse [8]. Il ressort que des antibiotiques habituellement prescrits, aucun germe isolé ne présente de sensibilité à l'amoxicilline, aucun des germes ne dépasse 50% de sensibilité aux ceftriaxone, ciprofloxacine alors que plus de 80% de souches d'*E. coli et Salmonella Spp.* sont sensibles au cotrimoxazole. La faible sensibilité des germes isolés à la plus part des antibiotiques habituellement prescrit plaide en faveur de la mise en place d'un système de surveillance épidémiologique pour orienter périodiquement et par zone l'antibiotique à prescrire quand il est suspect qu'une bactérie soit la cause d'une diarrhée. Étant donné qu'il est probable que cette sensibilité varie en fonction des souches des différents germes qui peuvent aussi varier par zone géographique, par groupe d'âge et par saison.

Le plateau technique et l'accès aux ressources à l'équipe qui a conduit la présente étude était assez limitée pour permettre l'investigation des causes les plus fréquentes de diarrhée que sont les virus, des diarrhées systémiques. Il serait nécessaire de conduire cette étude sur une année complète dans l'espoir de faire le profil annuel des germes associés aux bactéries et de leur sensibilité. Dans les mêmes conditions, il n'était pas possible que l'équipe de recherche se prononce avec certitude sur la relation de causalité qu'il y avait entre les germes isolés et les symptômes de diarrhée rapporté par le patient. L'étude a bien montré que la surveillance du profil des bactéries responsables de diarrhée ainsi que de leur sensibilité aux antibiotiques est faisable dans un contexte de ressources limitées.

## Conclusion

Au cours de la période ciblée et à l'hôpital régional annexe de Kousseri, deux cas de diarrhées sur 3 sont associés à la présence dans les selles d'une bactérie entéropathogènes. *Eschérichia coli, Salmonelles, Shigella Spp., Vibrio Spp. et Aeromonas sobria* constituent les bactéries pathogènes isolés. La proportion de ceux de ces bactéries sensibles aux antibiotiques fréquemment prescrite est très basse. Parmi les antibiotique frequemment prescrit, la ciprofloxacine reste le plus sensible aux entéropathogènes suivie de la ceftriaxone, la gentamicine et de l'amikacine. Au vu de ces résultats, il peut être admis qu'un système de surveillance des germes associés aux diarrhées et de leurs profils de sensibilité est faisable dans le contexte de l'hôpital régional annexe de Kousseri. Nous recommandons: Aux autorités du ministère de la santé publique de mettre en place un système de surveillance sentinelle des germes responsables des diarrhées et du profil de sensibilité de ses germes dans les hôpitaux du niveau régional; aux administrateurs des formations sanitaires de mettre à jours les protocoles de prise en charge des diarrhées en prenant en compte les résultats de la présente étude; à la communauté scientifique d'étendre la présente étude à un an afin qu'elle puisse capter la variation de distribution des germes en fonction des saisons. Inclure dans les systèmes de surveillance à mettre en place, un système d'investigation des souches isolées et de la causalité entre les germes isolés dans les échantillons de selles des patients diarrhéiques et le symptôme de diarrhée rapporté par le patient.

### Etat des connaissances actuelle sur le sujet

- La diarrhée est le symptôme de diverses infection causée par les bactéries, les virus ou des parasites;
- Au Cameroun, la prise en charge dans certaines formations sanitaires repose sur un diagnostic de présomption;
- De même, il y a très peu voir quasiment pas d'étude qui traitre de l'étiologie bactérienne de la diarrhée encore moins de la surveillance de la sensibilité aux ATB des bactéries associées à ces diarrhées.

### Contribution de notre étude à la connaissance

- Description du profil de bactéries pathogènes en circulation dans la région de l'extrême nord Cameroun;
- Description de la sensibilité aux antibiotiques des bactéries enteropathogénes responsables des diarrhées dans la localité;
- Vulgariser l'efficience de la mise en place d'un système de surveillance de la sensibilité aux ATB des bactéries pathogènes responsable des diarrhées dans les hôpitaux du niveau de références au Cameroun.

## Conflits d’intérêts

Les auteurs ne déclarent aucun conflit d'intérêts.

## Remerciements

Cette étude a été réalisée grâce au soutien financier et logistique de l'ONG Meilleur Accès aux Soins de Santé(MASANTE). Nous tenons à remercier l'administration de l'Hôpital Régional annexe de Kousseri(HRAK), pour avoir favorisé la mise en œuvre de ce projet. Un merci particulier au Dr Frank C. GUIMFACK pour avoir éditer le présent travail.
